# Long-term outcomes of sentinel lymph node navigation surgery for early-stage cervical cancer

**DOI:** 10.1007/s10147-024-02605-0

**Published:** 2024-09-02

**Authors:** Shinichi Togami, Nozomi Furuzono, Mika Mizuno, Shintaro Yanazume, Hiroaki Kobayashi

**Affiliations:** Department of Obstetrics and Gynecology, Faculty of Medicine, Kagoshima University, Sakuragaoka 8-35-1, Kagoshima, 890-8520 Japan

**Keywords:** Cervical cancer, Prognosis, Sentinel lymph node, Sentinel node navigation surgery

## Abstract

**Background:**

Sentinel lymph node navigation surgery, which identifies the sentinel lymph node in early cervical cancers and omits systemic pelvic lymphadenectomy in cases where no lymph node metastasis is present, has recently gained attention. However, there are few reports on lymph node recurrence and the long-term outcomes of cervical cancer surgery performed using sentinel lymph node navigation surgery. In this study, we aimed to evaluate the long-term outcomes of sentinel node navigation surgery for early-stage cervical cancer.

**Methods:**

One hundred thirty-eight patients with cervical cancer were enrolled. Sentinel lymph nodes were identified by injecting 99 m Technetium-labeled phytate and indocyanine green into the uterine cervix. Surgery and survival outcomes were also analyzed.

**Results:**

The median age and body mass index of the patients were 40 years (20–78) and 21.7 kg/m^2^ (16.5–50.4), respectively. Open surgery, laparoscopic surgery, and robotic surgery were performed in 77 (56%), 53 (38%), and 8 (6%) patients, respectively. The overall and bilateral detection rates of the sentinel lymph node were 100% and 94%, respectively. Only one case (0.7%) exhibited lower extremity lymphedema, and pelvic lymphocele was observed in three cases (2.2%). Four cases (3%) experienced recurrence over a median follow-up of 57.5 months (range, 2–115 months), with five-year recurrence-free and overall survival rates of 97% and 97.3%, respectively.

**Conclusions:**

Our results demonstrate that sentinel node navigation surgery may be safe and effective for early-stage cervical cancer.

## Introduction

The presence of pelvic lymph node metastasis (LNM) in cervical cancer is a crucial prognostic factor [1, 2]. Radical hysterectomy, including pelvic lymphadenectomy (PLA), is the standard procedure [3, 4]. However, intraoperative and postoperative complications attributed to PLA, such as vascular and nerve injuries, lower extremity lymphedema (LEL), pelvic lymphocele, and intestinal obstruction, can lead to a decline in the patient's quality of life (QOL) [5]. In particular, lymphatic complications such as LEL and pelvic lymphocele not only reduce QOL due to changes in the appearance of the lower extremities, numbness, and pain but also warrant attention as they may be associated with cellulitis [6].

According to previous reports, the incidence of LEL after PLA ranges from 10 to 40%, with the removal of inguinal lymph nodes considered a significant risk factor [5, 7–11]. In contrast, the incidence of pelvic lymphoceles is between 10 and 25% [8, 12–14], mostly occurring asymptomatically in the early postoperative period. However, if accompanied by infection, surgical interventions, such as percutaneous drainage, are necessary.

Sentinel lymph node (SN) biopsy for cervical cancer not only reduces postoperative LEL and pelvic lymphocele but also enables accurate diagnosis of LNM. A large prospective study showed high SN detection rates, sensitivity, and false negatives in patients with cervical cancer, suggesting that SN biopsy could be an alternative to systematic PLA [15]. Recently, SN navigation surgery (SNNS), which identifies the SN in early cervical cancers and omits systemic PLA in cases where no LNM is present, has gained attention. However, there are few reports on lymph node recurrence and the long-term outcomes of cervical cancer surgery performed using SNNS. In this study, we aimed to estimate the incidence of lymph node recurrence and long-term outcomes in patients with early-stage cervical cancer.

## Materials and Methods

### Patients

From December 2014 to February 2023, surgeries involving the use of SNNS in conjunction with radical/modified radical hysterectomy or radical trachelectomy were performed in 138 patients with stage IA2–IB1 cervical cancer at the Obstetrics and Gynecology Department of XX University Hospital. This study was approved by the institutional ethics committee, and informed consent was obtained from all patients. Staging according to the International Federation of Gynecology and Obstetrics (FIGO) criteria (2008) was determined based on clinical examinations, preoperative computed tomography, and magnetic resonance imaging. The inclusion criteria for the study were delineated as follows: (1) confirmation of cervical cancer via biopsy; (2) pre-surgical histology indicating squamous cell carcinoma, adenocarcinoma, or adenosquamous carcinoma; and (3) a maximum tumor diameter of less than 3 cm. Exclusion criteria encompassed patients with deep cervical stromal invasion, suspected peritoneal dissemination, or LNM.

### SN identification

SN identification was performed using the hybrid radioisotope fluorescence method. On the day prior to surgery, 1 mL of 99mTc-labeled phytate (4.0 mCi) was injected into the four quadrants of the cervix. Subsequently, the location of the SN was estimated using lymphoscintigraphy and single-photon emission computed tomography/computed tomography. Immediately before surgery, indocyanine green diluted tenfold was injected into the cervix at the 3 and 9 o’clock positions. During the operation, SN identification was conducted using a gamma probe (Neoprobe Corporation, Dublin, OH, USA) and a near-infrared light endoscopic camera (SPIES, Karl Storz Endoscopy, Japan). SN removal was diagnosed by intraoperative rapid diagnosis, and systematic PLA was omitted if LNM was negative. Ipsilateral PLA was performed if SN identification was not possible. Regarding the intraoperative diagnosis of SN metastasis, the excised SN was sectioned at 2 mm intervals using the short-axis method, and the presence of metastasis was diagnosed histopathologically. February 2021 onwards, the excised SN was diagnosed with metastasis using the OSNA method. SNs diagnosed intraoperatively by histopathological methods were finally diagnosed with HE staining of permanent specimens; however, ultra-staging was not performed. Regarding the diagnosis of SN metastasis, SNs were considered metastasis-positive if micrometastasis or macrometastasis was present. The OSNA method was diagnosed as positive and negative when the CK19 mRNA copy number was greater than 250 cCP/μL and less than 250 cCP/μL, respectively. There were no cases in which SNs diagnosed as metastasis-negative intraoperatively were diagnosed as metastasis-positive in permanent specimens.

### Adjuvant therapy

Postoperative adjuvant therapy was administered to patients presenting with tumors larger than 4 cm, a depth of cervical stromal invasion exceeding one-half, and evidence of positive LNM as confirmed by postoperative pathological examination.

### Evaluation of lymphatic complications

A gynecologic oncologist collected patient information at each follow-up outpatient visit. LEL was diagnosed according to the International Lymphatic Society guidelines [16]. Conversely, pelvic lymphocele was assessed through transvaginal ultrasound and computed tomography. The evaluation was conducted in accordance with the Common Terminology Criteria for Adverse Events version 4.0.

### Statistical analysis

Relapse-free survival (RFS) and overall survival (OS) were analyzed using Kaplan–Meier curves. JMP software (SAS Institute Inc., Cary, NC, USA) was used for statistical analyses. *P* < 0.05 was considered statistically significant.

## Results

The clinicopathological characteristics of the 138 cases of cervical cancer with SNNS are presented in Table 1. The median ages and body mass indices of the patients were 40 years (20–78) and 21.7 kg/m^2^ (16.5–50.4), respectively. The final histological types were squamous cell carcinoma, adenocarcinoma, adenosquamous cell carcinoma, and others, with 99 (72%), 31 (22%), 3 (2%), and 5 (4%) cases, respectively. FIGO stages (2008) were IA2/IB1/IB2/IIB, comprising 23 cases (17%)/111 cases (80%)/1 case (1%)/3 cases (2%), respectively. Open surgery, laparoscopic surgery, and robotic surgery were performed in 77 (56%), 53 (38%), and 8 (6%) patients, respectively. The median operation time, blood loss, and length of hospital stay were 276 min (169–741), 240 g (169–741), and 6 days (4–14), respectively. Adjuvant therapy included chemotherapy in nine cases (7%) and radiotherapy in 17 cases (12%), with four cases (3%) showing recurrence; however, no lymph node recurrence was observed.

**Table 1:** Clinicopathological characteristics

	N = 138
Median age (years)	20–78 (40)
Median BMI (kg/m^2^)	16.5–50.4 (21.7)
Final pathology	
SCC	99 (72%)
Adenocarcinoma	31 (22)
Adenosquamous cell carcinoma	3 (2)
Other	5 (4)
FIGO staging (2008)	
IA2	23 (17%)
IB1	111 (80)
IB2	1 (1)
IIB	3 (2)
Surgical approach	
Open	77 (56%)
Laparoscopy	53 (38%)
Robot	8 (6%)
Median operation time, min (range)	276 (169–741)
Median blood loss, g (range)	240 (5–1520)
Median length of hospital stays, days (range)	6 (4–14)
Adjuvant therapy	
None	112 (81%)
Chemotherapy	9 (7)
Radiotherapy	17 (12)
Recurrence	
No	134 (97%)
Yes	4 (3)

*BMI* body mass index, *SCC* squamous cell carcinoma, *FIGO* international federation of gynecology and obstetrics

**Fig. 1:**
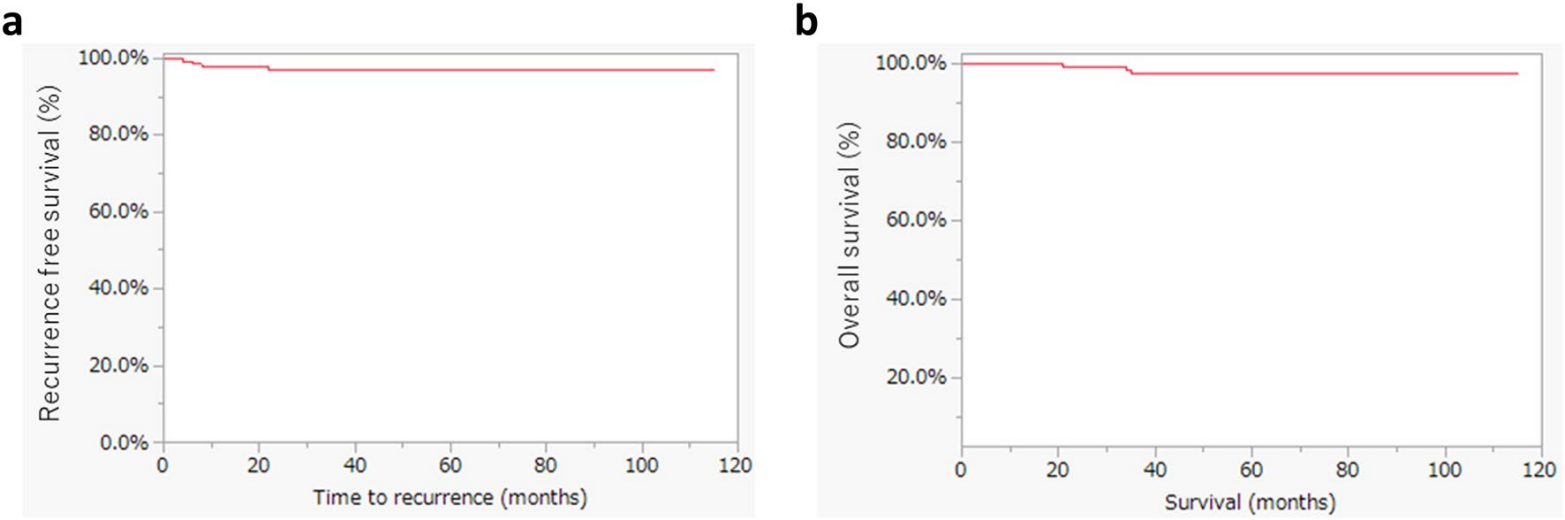
**a** Five-year recurrence-free survival rate; **b** five-year overall survival rate

The outcomes of SN removal are shown in Table 2. SN identification was performed in 138 cases, with a 100% detection rate (at least one SN identified); the bilateral pelvic SN identification rate was 94% (130/138). A total of 288 SNs were identified, and their locations were obturator, external iliac, internal iliac, common iliac, and parametrial in 141 (49%), 123 (42%), 17 (6%), 6 (2%), and 1(1%), respectively. The median number of SNs removed per case was two (range, 1–5), and SN metastasis was observed in four cases (2.9%), with two cases of macrometastasis and two cases of micrometastasis. Among the 134 cases in which PLA was omitted, only one (0.7%) exhibited LEL. Pelvic lymphocele was observed in three cases (2.2%). Four patients (3%) experienced recurrence during a median follow-up period of 57.5 months (range, 2–115 months). The sites of recurrence were the vaginal stump in two cases, pelvic mass in one case, and lung metastasis in one case; three of them died, and the remaining one survived with no evidence of disease (Table 3). Furthermore, using the Kaplan–Meier method for 138 cases of cervical cancer with SNNS, the estimated 5-year RFS and OS rates were 97% and 97.3%, respectively (Fig. 1), indicating favorable outcomes.

**Table 2:** Sentinel lymph node-related outcomes

	Patients (N = 138)
SLN mapping	
Bilateral	130 (94%)
Unilateral	8 (6%)
SLN locations (n = 288)	
Obturator	141 (49%)
External iliac	123 (42%)
Internal iliac	17 (6%)
Common iliac	6 (2%)
Parametrial	1 (%)
Median number of SLN removed (range)	2 (1–5)
SLN metastasis	4 (3%)
Lower extremity lymphedema	1 (1%)
Pelvic lymphocele	3 (2.2%)

SLN: sentinel lymph node

**Table 3:** Details of the recurrence cases

	Age	FIGO staging (2008)	Surgery	Final pathology	Pathological tumor size (mm)	Adjuvant therapy	SN metastasis	Recurrence site	Time to recurrence (months)	Status
Patient 1	34	IB1	Open	SCC	20	None	None	lung	8	DOD
Patient 2	43	IB1	Laparoscopy	SCC	26	None	None	vaginal stump	4	DOD
Patient 3	34	IB1	Laparoscopy	SCC	25	None	None	vaginal stump	6	NED
Patient 4	57	IB1	Laparoscopy	Adenocarcinoma	25	None	None	Pelvic mass	22	DOD

*FIGO* International Federation of Gynecology and Obstetrics, *SN* sentinel lymph node, *SCC* squamous cell carcinoma, *DOD* dead of disease, *NED* no evidence of disease

## Discussion

SN mapping for cervical cancer is gradually becoming more widespread in Japan [17] and is described as a standard procedure in overseas guidelines [18]. This study investigated the effect of SNNS on the long-term prognosis of patients with cervical cancer. In this study, no lymph node recurrence was observed in patients with SNNS, and the prognosis was good, with RFS and OS rates of 96.4% and 96.6%, respectively. Therefore, SNNS for cervical cancer is safe and effective without increasing recurrence.

In this study, SNNS was performed for cervical cancer cases of less than 3 cm, and 56% of cases were operated on by laparotomy, partly due to the worldwide decrease in minimally invasive surgeries such as laparoscopy and robotics, according to the results of the LACC study [19]. However, the most significant reason for this is that in Japan, laparoscopic radical hysterectomy is restricted to tumors smaller than 2 cm, and robotic surgery is not yet covered by insurance.

In our previous study [20], the SN detection rate (at least one SN identified) in 70 patients who underwent SNNS was 100%. In the SENTICOL study [15], SNs were detected in 136 (97.8%) of the 139 patients (95% confidence interval, 93.8–99.6%), and SNs were found on both sides in 104 (76.5%) patients. Contrarily, Aoki et al. [21]. identified SN using ICG in laparoscopic surgery for cervical cancer, and the per-patient and side-specific SN detection rates were 98.7% (76/77 patients) and 93.5% (144/154), respectively. In the present study, the detection rate of SNs (at least one SN identified) was 100%, and the bilateral SN identification rate was 94% (130/138). Consistent with previous reports, the SN identification rate in cervical cancer appears to be high. In this study, SN metastasis was observed in four cases, with two cases of macrometastasis and two cases of micrometastasis. Regarding micrometastasis, there is a possibility of poor prognosis, and a prospective clinical trial is currently underway, with clear evidence for SNNS still awaited [22, 23].

Previous studies have reported that the incidence of LEL with PLA in patients with cervical cancer is between 16.6% and 42% [5, 7–11]. In contrast, the LEL occurrence rate in patients who underwent SNNS for cervical cancer was reported to be 0–5.6% [20, 24, 25]. In the present study, the incidence of LEL in patients in whom PLA could be omitted with SNNS was 0.7%, suggesting that SNNS may improve patient QOL, which is similar to results from previous reports. In contrast, Yahata et al. reported a low incidence of pelvic lymphocele at 0.6% in their study, which focused on SNNS cases of cervical cancer [25]. Here, we also found that only 2.2% of patients without PLA had a pelvic lymphocele, suggesting that SNNS can reduce lymphatic-related complications.

Lennox et al. [26] reported that recurrence occurred in four patients with SNNS, and the five-year RFS was 93%. Favre et al. [27] reported that the four-year disease-free survival (DFS) and OS rates of 105 patients with SNNS were 89.5% and 95.2%, respectively. In this study, four cases (3%) experienced recurrence over a median follow-up period of 57.5 months. The 5-year RFS rate and OS rate were 97% and 97.3%, respectively.

Our study had a longer median follow-up than these reports did, and the RFS and OS were more favorable.

In contrast, Balaya et al. [28] reported that there were 10 recurrences (11.5%) in 87 patients with SNNS, and the DFS was 85.1% (median follow-up, 47 months). Minimally invasive surgery was performed in 95.4% of the patients, and approximately 10% of the cases were high-risk with tumor diameters greater than 2 cm in diameter, which may have contributed to a worse prognosis. Yahata et al. [24] reported on the long-term follow-up of 181 SNNS cases, observing four instances of recurrence. The 5-year progression-free survival and OS rates were noted as 98.8% and 99.4%, respectively. These results indicate that SNNS is safe with respect to long-term prognosis, which is similar to the outcomes of our study.

This study had several limitations. First, this was a retrospective study, which inherently carries a risk of selection bias. Second, although this study analyzed the long-term prognosis of 138 cases of cervical cancer SNNS, the number of cases remains limited. Future research with a larger study population is required to validate our findings.

In conclusion, our data demonstrated that SNNS is an effective and safe procedure for patients with early-stage cervical cancer. A large prospective trial on SNNS to evaluate the prognosis of early-stage cervical cancer is warranted.
